# A Unique Ru-N_4_-P Coordinated Structure Synergistically Waking Up the Nonmetal P Active Site for Hydrogen Production

**DOI:** 10.34133/2020/5860712

**Published:** 2020-08-28

**Authors:** Chuanqiang Wu, Shiqing Ding, Daobin Liu, Dongdong Li, Shuangming Chen, Huijuan Wang, Zeming Qi, Binghui Ge, Li Song

**Affiliations:** ^1^National Synchrotron Radiation Laboratory, CAS Center for Excellence in Nanoscience, University of Science and Technology of China, Hefei, Anhui 230029, China; ^2^Institutes of Physical Science and Information Technology, Anhui University, Hefei 230601, China; ^3^Institute of Amorphous Matter Science, School of Materials Science and Engineering, Hefei University of Technology, Hefei, Anhui 230009, China; ^4^Experimental Center of Engineering and Material Science, University of Science and Technology of China, Hefei 230026, China

## Abstract

Numerous experiments have demonstrated that the metal atom is the active center of monoatomic catalysts for hydrogen evolution reaction (HER), while the active sites of nonmetal doped atoms are often neglected. By combining theoretical prediction and experimental verification, we designed a unique ternary Ru-N_4_-P coordination structure constructed by monodispersed Ru atoms supported on N,P dual-doped graphene for highly efficient hydrogen evolution in acid solution. The density functional theory calculations indicate that the charge polarization will lead to the most charge accumulation at P atoms, which results in a distinct nonmetallic P active sites with the moderate H∗ adsorption energy. Notably, these P atoms mainly supply highly efficient catalytic sites with ultrasmall absorption energy of 0.007 eV. Correspondingly, the Ru-N_4_-P demonstrated outstanding HER performance not only in an acidic condition but also in alkaline environment. Notably, the performance of Ru-NPC catalyst at high current is even superior to the commercial Pt/C catalysts, whether in acidic or alkaline medium. Our in situ synchrotron radiation infrared spectra demonstrate that a P-H_ads_ intermediate is continually emerging on the Ru-NPC catalyst, actively proving the nonmetallic P catalytically active site in HER that is very different with previously reported metallic sites.

## 1. Introduction

Recently, single-atom catalysts (SACs) are synthesized by doping active atoms onto the surface of a catalyst. These active atoms provide more active catalytic centers, consequently enhancing the catalyst's catalytic performance [1–15]. In particular, monoatomic catalysts were used as electrochemical catalysts for hydrogen production to replace Pt-like precious metal catalysts [16–21]. It was used as a favorable system for a catalytic reaction because of the homogeneous structure of active sites and optimum utilization of the active center atom. Hence, finding suitable metal and nonmetal catalysts and optimizing the use of catalysts enhancing activity toward HER are essential [22–24].

The development of a monoatomic catalyst, carbon nanomaterial is widely used as an excellent support to anchor the single active atoms by the assistance of the intrinsic defects, heteroatoms, and functional groups, which can effectively lower their cohesive energies to prevent the formation of metal aggregations [25–28]. In particular, metal-Nx coordinated carbon nanostructures have been extensively demonstrated as the catalytically active sites to enhance the catalytic performance for various reactions rather than the presence of nanoparticles [24–30]. In this way, the nonmetallic atoms change the electronic structure of the bonded metals to improve their catalytic performance. Such nonmetallic elements may become anomalous active centers due to the simultaneous interaction from metal elements. Therefore, it will be very exciting to study nonmetallic catalysts with a monoatomic structure. For example, Qiao et al. reported a nonmetal iodine single-atom electrocatalyst with robust structural stability and exceptional electrocatalytic activity for HER in an alkaline solution [31]. Lin et al. group-fabricated a graphene-analogous material, in which the active carbon atom is displaying unusual activity toward the HER under acidic conditions [32]. Works in this direction are still at an early stage; there is still a significant challenge in understanding the catalytic reaction mechanism in monoatomic nonmetal catalysts. Consequently, the exploration of new catalysts with nonmetallic catalytic sites is highly desirable, especially for the application in different pH chemical environments.

Herein, combining density functional theory (DFT) calculations with the structural characterizations, we constructed a unique Ru-N_4_-P structure in graphene (Ru-NPC). The Ru atom can act as a donor to provide electrons while P mainly supplies a high efficient catalytic site with ultrasmall absorption energy. Due to the synergistic effect between P and Ru, the as-prepared Ru-NPC catalysts exhibited superior HER performance than the control Ru-NC or commercial Pt/C catalysts at acid or alkaline environment. This unique engineering and the coordination environment of well-dispersed monoatomic structures with metal and nonmetal bonding provide a better understanding of synergistic effect in SACs, thus opening a new way for the future rational design of highly efficient HER catalytic materials with multiple active sites.

## 2. Results and Discussions

### 2.1. Theoretical Prediction

At present, numerous efforts have been devoted to developing highly efficient catalysts for HER to replace precious metal catalysts. Among them, ruthenium monoatomic catalysts anchored by nitrogen atoms (Ru-N_4_ structure) were considered a better choice for monoatomic catalysts. However, the performance of Ru monoatomic catalysts still has much room for improvement. Hence, we designed a P atom-doped Ru single-atom catalyst supported on N-doped graphene for HER in acid solution. Meanwhile, we have carried out in-depth density functional theory (DFT) calculations.

**Figure 1:**
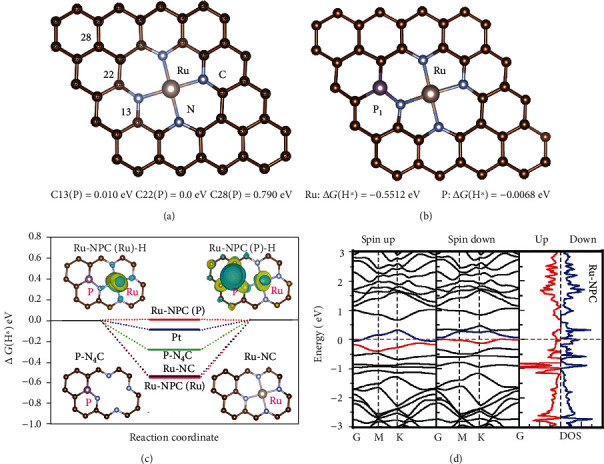
(a) The total energy of the Ru-N-P system when the P atom occupies in different positions compared with C22 (P). (b) Gibbs free energy of Ru-NPC at Ru and P_1_ sites. (c) The calculated Gibbs free energy diagram of the HER at the equilibrium potential for Pt, P-N_4_C, Ru-NC, Ru-NPC (Ru), and Ru-NPC (P). The inset pictures are the structure of P-N_4_C, Ru-NC, and differential charge density of Ru-NPC (Ru) and Ru-NPC (P). (d) The calculated band structure (left) and density of states (right) of Ru-NPC.

First of all, we calculated the structural stability of the P atom doped into the Ru-N_4_ system. We consider that the total energy of the system is the P atom occupying different positions. We found that if the P atom locates on the farther position from the Ru-N_4_ structure, the system will become more unstable with higher total energy. Even though C22 and C13 are very close to Ru atoms, they have different structures and belong to different positions. As shown in Figure 1(a), when P atoms occupy C22 positions, the system's total energy is the lowest and the structure is the most stable, indicating the doping P very likely to occupy the C22 position to form a Ru-N_4_-P structure. After determining the atoms' occupation structure, we calculated the atoms' hydrogen production activity at different positions. It is remarkable that the Gibbs free energy of hydrogen adsorption (Δ*G*(H∗)) on the surface strongly affects the HER activity of the catalyst. An excellent electrocatalyst for HER usually has an optimal value being close to zero. Due to P atoms having two possible different positions occupied in the second coordination shell of Ru atoms, three models of Ru-NC, Ru-NP_1_C and Ru-NP_2_C, were calculated, respectively (Figure S1 and Figure 1(b)). The calculation results revealed that the Ru active site in three models has the Δ*G*(H∗) value of -0.534 eV, -0.575 eV, and -0.551 eV. Obviously, in the Ru-N_4_ structure, the adsorption free energy of the Ru atom is enormous, which is not conducive to catalytic hydrogen production. At the same time, the absolute value of Δ*G*(H∗) for the Ru atom increases slightly after doping P atom, indicating that the catalyst activity has not been significantly improved.

Surprisingly, when we consider the catalytic activity of the surrounding coordination atoms, we get unexpected results. As shown in Figure 1(c), the most favorable active site of Ru-NPC is the P_2_ atom with an ultrasmall Δ*G*(H∗) value of only 0.007 eV, closer to zero than the P_1_ atom with -0.359 eV and Pt catalyst. These results suggested a mediated adsorption-desorption behavior of Ru-NPC to facilitate the overall HER performance. Besides, the system will have the lower total energy for Ru-NP_1_C than Ru-NP_2_C. The investigation of their electronic structures further demonstrated the advantage of the P atom for HER. When H attaches to the P atom or the Ru atom, the electron structure changes significantly. More interestingly, there are a higher number of positively charged on the P atom compared with the Ru atom in Ru-NPC (+0.88e vs. +0.01e). It results in more significant hydrogen adsorption to facilitate the overall HER performance on the P atoms in Ru-NPC. Furthermore, in the absence of Ru atoms, the P-N_4_C structure has an undesirable Δ*G*(H∗) of the P atom. We also calculated the absorption energy of H∗ absorbed on N sites. However, the absorbed H∗ will migrate and bond to the Ru atom after optimization. Therefore, it can be concluded that the superior HER performance comes from the biactive site and synergistic effect of Ru and P atoms in the unique Ru-N_4_-P structure. It is well known that the conductivity of catalysts is a critical factor affecting the electrocatalytic performance.  Figure 1(d) and Figure S2 show the calculated band structure and density of states of P-doped graphene, Ru-NC, and Ru-NPC, respectively. It was seen that the P-doped graphene is a semiconductor material with a large bandgap of about 1 eV which means weak electrical conductivity limits the HER. In contrast, the Ru-NC and Ru-NPC are both metallic, which means the introduction of Ru atoms should bring in many charge carriers.

### 2.2. Synthesis and Morphology Characterization of Ru-NPC

**Figure 2:**
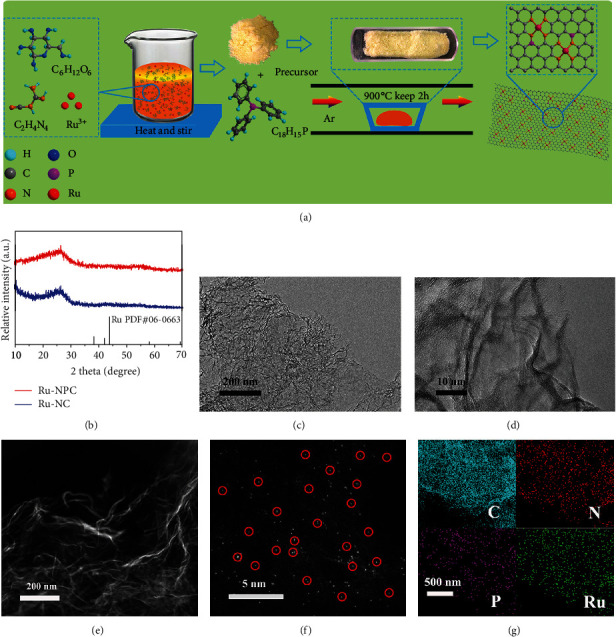
(a) Schematic diagram of the synthesis steps of a typical sample of Ru-NPC. (b) XRD patterns of Ru-NPC and Ru-NC compared with standard card PDF#37-1492. (c–e) The TEM, HRTEM, and HAADF-STEM images of Ru-NPC show a typical two-dimensional graphene structure with no impurity particles on the surface. (f) The local high-resolution STEM image shows that Ru-NPC displays a random dispersion of Ru single atoms on the nanosheets. (g) The EDS mapping proved that atoms of C, N, P, and Ru are uniformly distributed among the Ru-NPC sample.

Our simulation results hint that on successfully synthesizing the Ru-NPC material, we will get an excellent electrocatalytic HER catalyst acid solution. As illustrated in Figure 2(a), the preparation of atomically dispersed Ru-NPC catalysts utilized an in situ confined pyrolysis process. Experimentally, the triphenylphosphine was added as the phosphorus source to pyrolyze at an elevated temperature, apart from glucose, dicyandiamide, and Ru^3+^ salt. Ru single atoms were trapped by the heteroatom anchoring sites and stabilized evenly on the surface of N,P-codoped graphene supports. X-ray diffraction (XRD) was carried out to probe the crystal structure and phase of the obtained materials.  Figure 2(b) shows the XRD patterns of Ru-NC and Ru-NPC compared with Ru metal standard card (PDF#06-0663). Ru-NC and Ru-NPC display a broad diffraction peak around 26°, which correspond to crystalline carbon structures, with no diffraction peaks for Ru metal and compounds. A scanning electron microscope (SEM) was used to analyze the overall morphology of the material. Figures S1-S4 show SEM images and EDS mapping of NC, NPC, Ru-NC, and Ru-NPC. Interestingly, all the sample displays a soft and loose structure which consists of crinkly nanosheets. The crinkly structure indicates its ultrathin thickness. Also, it reveals that the morphology can keep the original appearance of graphene nanosheets after doping Ru atoms and the elements are evenly distributed throughout the sample. Figure S7 shows that all the samples have a high specific surface area.

The microstructures of samples were further investigated utilizing transmission electron microscopy (TEM) and scanning transmission electron microscopy (STEM).  Figure 2(c) shows the TEM image of a typical sample of Ru-NPC; it reveals that the sample is composed of ultrathin nanosheets. The corresponding high-resolution TEM in Figure 2(d) displays an apparent layered structure of graphene nanosheets. To confirm the presence of Ru single atoms in the sample, we employed the subangstrom resolution high-angle annular dark-field scanning transmission electron microscopy (HAADF-STEM) for Ru-NPC. As shown in Figure 2(e), the material exhibits a typical soft two-dimensional graphene structure with no impurity particles on the surface. Moreover, the local high-resolution STEM image (Figure 2(f)) shows that Ru atoms are uniformly dispersed in the plane. Figure S8 shows more images illustrating the dispersion of individual Ru single atoms. Furthermore, the electron dispersive spectroscopy (EDS) mapping suggests the homogeneous distribution of C, N, P, and Ru atoms over the entire nanosheet (Figure S8c and Figure 2(g)). The Ru content is estimated to be about 1.25 wt%, measured by EDS analysis. The analysis of compared sample Ru-NC is showed in Figure S9, which exhibits similar structural characteristics and element distribution with the Ru-NPC sample. Therefore, it can be concluded that N,P dual-doped graphene supporting Ru single atoms were successfully synthesized.

### 2.3. Structural Characterization and Verification

In order to characterize the structure of the samples, we synthesized and verified the rationality of our calculation results; we carried out a series of tests. X-ray photoelectron spectroscopy (XPS) was used to study the detailed elemental composition, content, and chemical state of materials. The XPS results of NC, NPC, and Ru-NC are showed in Figures S10-S12. The fitting result of Ru-NPC is shown in Figure S13; the C 1s spectrum displays four peaks at 284.7, 285.7, 286.6, and 290.5 eV attributed to C=C, C-N/C-O/C-P, C=N/C=O, and O-C=O, respectively. The peak at 280.1 eV is attributed to Ru 3d binding energy. The N 1s spectrum can be divided into four types of environments, corresponding to the pyridinic-N (398.2 eV), pyrrolic-N (399.2 eV), graphitic-N (400.9 eV), and oxidized-N (403.2 eV). In addition, the peak at about 400.9 eV not only corresponds to graphitic-N and represents the N-P bonding. The high-resolution P 2p spectrum reveals two types of chemical bonding: P-C and P-N bonding at 132.5 eV and 133.5 eV [33, 34]. The P-N bonding is consistent with N-P bonding in the N 1s spectrum. Furthermore, the Ru 3p_3/2_ and Ru 3p_1/2_ peaks of Ru-NPC appear at a binding energy of 461.8 and 484.0 eV, respectively [6, 35]. According to fitting curves, only one Ru species exists in the Ru-NPC sample. The binding energy is higher than that of metallic Ru^0^ and close to Ru^2+^[3, 36]. This is consistent with the formation of Ru atomic species embedded within the carbon matrix, as manifested in TEM measurements. As shown in Figure S14a, the XPS curves of Ru-NC and Ru-NPC exhibit no significant change, indicating that they have similar binding energy and microstructure.

**Figure 3:**
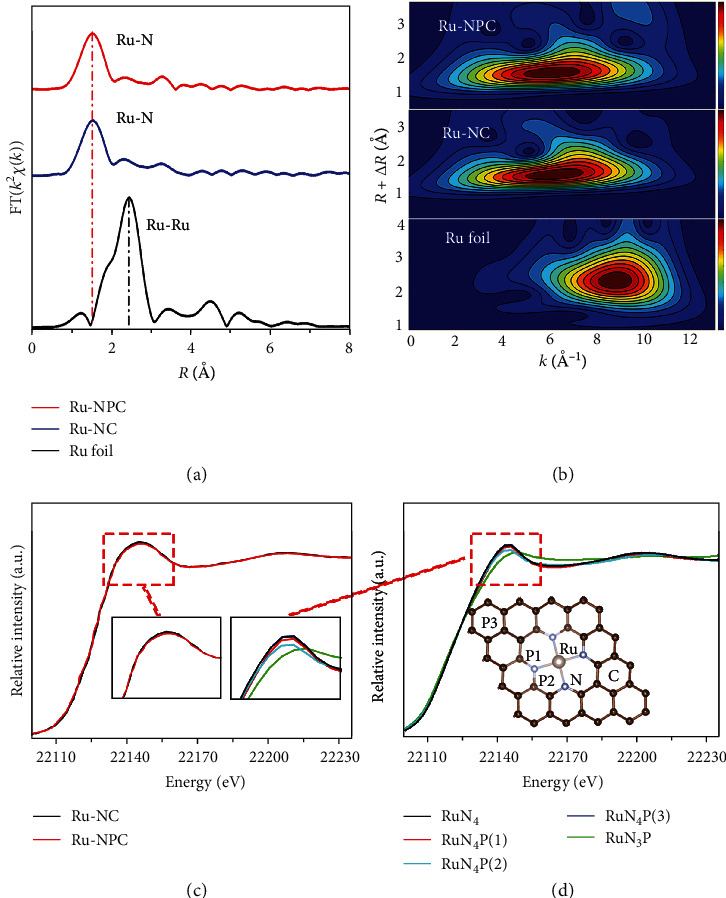
(a) Fourier transform (FT) of the Ru K-edge in Ru-NC, Ru-NPC, and Ru foil. (b) Wavelet transform (WT) of Ru-NC, Ru-NPC, and Ru foil, respectively. (c) The XANES spectra of Ru-NC and Ru-NPC. (d) The theoretical calculation of the XANES spectra with different structures.

To further investigate the electronic and coordination structures of Ru-NC and Ru-NPC, we performed the X-ray absorption near-edge spectroscopy (XANES) and extended X-ray absorption fine structure (EXAFS) measurements. Figure S14b shows the Ru K-edge XANES profiles for Ru-NC, Ru-NPC, and Ru foil. The XANES spectrum of Ru-NPC is almost the same as that of Ru-NC, which means that the doping P atoms do not change the nearest neighboring geometric structure around central Ru atoms. In other word, the doping P atom must not locate on the first coordination shell around Ru. The EXAFS Fourier transforms and wavelet transforms are shown in Figures 3(a) and 3(b), respectively. The FT-EXAFS spectra show that Ru-NC has almost the same Ru-N peak position and intensity with Ru-NPC. It indicates that Ru-NC has the same first coordination shell with Ru-NPC, consistent with the XANES analysis result. Furthermore, it can be observed both from FT-EXAFS and WT-EXAFS that there is no Ru-Ru bond existing in Ru-NC and Ru-NPC, indicating the excellent dispersion of Ru single atoms [37]. From the EXAFS fitting, the coordination number of Ru-N in the first shell is estimated to be about 3.7 and 3.8 for Ru-NC and Ru-NPC, respectively (Figure S15 and Table S1). Therefore, it can be concluded that these Ru single atoms bond with four N atoms forming a Ru-N_4_ local structure [4, 11]. As there is many scattering information in the second or farther shell, it can hardly determine P atoms' accurate position in Ru-NPC. Therefore, we calculated the XANES spectra of different structures theoretically (Figures 3(c) and 3(d)). When the P atom is located in the first shell, the absorption spectrum has deviated, so we can discard this assumption. When P atoms are in the second shell, there are two kinds of positions. When the P atom occupies the P_1_ position, the absorption spectrum structure is closer to the original Ru-N_4_ structure, with a slight decrease in the white-line peak; at the P_2_ position, the white-line peak decreases significantly, which is inconsistent with the experimental data. However, when the P atom is far away from the Ru atom, the absorption spectrum does not change and coincides with the original structure. So, the P atom is more likely to occupy the P_1_ position. In addition, combined with the XPS analysis result, it shows the P atom bond with neighboring C and N atoms. This result is well consistent with our theoretical calculation results and well agreeing with the experimental observations. Thus, we can suggest that P atoms should occupy P_1_ position in the second coordination shell of the Ru atom, forming a unique ternary Ru-N_4_-P microstructure.

### 2.4. Verification of Electrocatalytic Hydrogen Production

**Figure 4:**
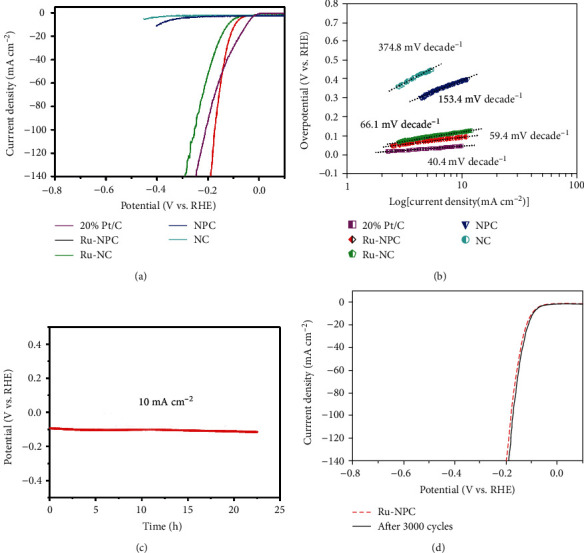
Comparative electrocatalytic hydrogen evolution of catalysts. (a) HER polarization curves of different samples and commercial 20% Pt/C catalyst in 0.5 M H_2_SO_4_ solution. (b) Corresponding Tafel plots of different samples and commercial 20% Pt/C catalyst in 0.5 M H_2_SO_4_ solution. (c) Time dependence of cathodic current density curve (*v*‐*t* curve) of Ru-NPC. (d) HER polarization curves of Ru-NPC before and after 3000 CV tests in 0.5 M H_2_SO_4_ solution.

The electrocatalytic properties were evaluated to verify the electrocatalytic hydrogen production performance of the P atom-doped Ru-N_4_ catalyst in an acid solution with a three-electrode work station. All polarization curves are not rectified for iR loss here.  Figure 4(a) shows the polarization curves of Ru-NPC, Ru-NC, NPC, NC, and 20% Pt/C. Notably, the Ru-NPC acts as the best HER catalyst compared to Ru-NC, NPC, and NC. The Ru-NPC catalyst requires quite small overpotentials of 93 mV to achieve current densities of 10 mA cm^−2^, which is very close to commercial 20% Pt/C. Especially under high current density, the Ru-NPC exhibited better performance than commercial 20% Pt/C, suggesting the Ru-NPC catalyst's excellent electrocatalytic activity. Also, NPC showed a significant performance improvement than NC after P doping. Therefore, it can be concluded that P atoms' doping into catalyst brings out a significant enhancement of the HER activity. The Tafel plots of the corresponding polarization curves are displayed in Figure 4(b). The Tafel slope of Ru-NPC is 59.4 decade^−1^, which is comparable to commercial 20% Pt/C catalyst. At the same time, the Ru-NPC is superior to other comparison samples. The comparisons of electrocatalytic HER activity about single-atom catalysts are shown in Table S2. To evaluate the stability of the catalysts, chronopotentiometry tests and constant-current technique were conducted.  Figure 4(c) shows that the overpotential remains nearly constant under continuous electrolysis reaction more than 20 h at a current density of 10 mA cm^−2^, and the LSV curves show no noticeable degradation in HER activities, further demonstrating the excellent stability and durability of the compound.  Figure 4(d) shows the LSV curves of Ru-NPC catalysts before and after CVs for 3000 cycles in acidic solutions. HAADF-STEM images (Figure S16) of the Ru-NPC catalyst after a long-term durability test show typical monoatomic characteristics, consistent with the sample before the reaction indicating the good stability of the catalyst. The active surface areas were analyzed through their electrochemical double-layer capacitances. Figures S15-S17 show CVs and determine electrochemically active surface areas of NC, NPC, Ru-NC, and Ru-NPC catalysts at different scan rates in acidic, alkaline, and neutral solutions. The Ru-NPC catalyst shows a comparable larger active area in all environments. What is surprising is that when we use this catalyst in an alkaline environment, it also has an excellent catalytic effect. Accordingly, the Ru-NPC catalyst requires quite small overpotentials of 78 mV to achieve current densities of 10 mA cm^−2^ in alkaline solutions. Besides, the Ru-NPC even exhibited better performance than commercial 20% Pt/C under high current density. The Tafel slope of Ru-NPC is 68.3 decade^−1^; meanwhile, it keeps good catalytic cycle stability (Figure S20).

### 2.5. Determination of the P Atom in Active Site

**Figure 5:**
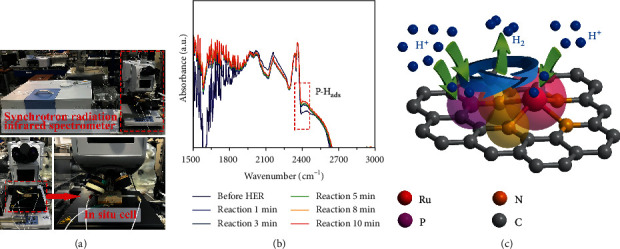
(a) The synchrotron radiation infrared spectrometer and in situ cell. (b) The in situ synchrotron radiation infrared spectra of Ru-NPC at different test times. (c) The catalytic reaction of P active site and Ru active site for HER.

In order to further confirm the proposed HER mechanism in the nonmetallic active site, we carried out in situ synchrotron radiation infrared spectroscopy to study the possible intermediate substances and role of active site P atoms in the reaction process (Figure 5(a)). As shown in Figure S21, there is no apparent infrared absorption of Ru-NC and Ru-NPC at about 2400 cm^−1^ in acid solution before HER which belongs to stretching vibration of P-H_ads_ absorption [38]. However, a distinct peak at about 2400 cm^−1^ is observed at a potential of -0.1 V (vs. RHE), suggesting that as HER reaction begins, the H atoms are adsorbed on the P atoms for Ru-NPC catalyst (Figure 5(b) and Figure S22). Meanwhile, with the increase of catalytic reaction time, the intensity of the absorption peak did not change, indicating that a P-H_ads_ intermediate continually forms during the HER. Correspondingly, there was no change in the IR spectra of the Ru-NC catalyst (Figure S23). Therefore, these observations confirm that the P atoms can accelerate water dissociation and H adsorption through P-H_ads_ bond formation, subsequently enhancing the HER kinetics. Combined with the theoretical calculation of the active site of Ru-NPC, we can finally conclude that the nonmetallic P atom is promoted as a new nonmetallic reactive center via the unique Ru-N_4_-P bonding structure, resulting in the significant improvement of HER performance (Figure 5(c)).

## 3. Conclusion

In summary, we designed a unique ternary Ru-N_4_-P structure for highly efficient hydrogen evolution according to theoretical calculation. The charge polarization can lead to the most charge accumulation at P atoms, which results in distinct nonmetallic P active sites with the moderate H∗ adsorption. Furthermore, the doped Ru contributed to the electrical conductivity while P mainly supplies a highly efficient catalytic site with ultrasmall absorption energy of 0.007 eV. Our HAADF-STEM, XAFS characterizations, and theoretical calculations reveal that the doped P atoms surround the RuN_4_ forming a unique Ru-N_4_-P structure. A promising achievement in this work, the Ru-N_4_-P catalyst, exhibits an excellent HER performance with small overpotentials of 93 and 78 mV at 10 mA cm^−2^ in acidic and alkaline solutions. A P-H_ads_ intermediate was continuously observed during the HER process by in situ synchrotron radiation IR spectra. This work not only supplies a competent candidate to substitute commercial Pt/C catalysts but also provides useful insight to clearly understand the HER mechanism in single-atom catalysts with metallic and nonmetallic active sites.

## 4. Materials and Methods

### 4.1. Experimental Method

Ruthenium (III) chloride (RuCl_3_), triphenylphosphine powder 99%, 5 wt% Nafion solution, and 20 wt% Pt/C were purchased from Alfa Aesar. Other chemical reagents were purchased from Sinopharm Chemical Reagent Co., Ltd. All chemicals were directly used without further purification. The water used in all experiments was deionized water (DIW). Argon gas used in this study was purchased from Nanjing Special Gas Factory Co., Ltd.

#### 4.1.1. N-Doped Graphene (NC)

Typically, 0.25 g glucose as the carbon source and 5 g dicyandiamide as the nitrogen source were dissolved in 200 mL deionized water to form a uniform solution. The solution was heated and stirred at 80°C, and the water was gradually evaporated to form a powder precursor. The precursor was put in a quartz crucible; then, the crucible was put in a tube furnace, annealed to 900°C at a rate of 3°C/min, and maintained in an Ar atmosphere for 2 hours. Finally, N-doped graphene was obtained by washing and ultrasonication of the sintered black powder.

#### 4.1.2. N,P-Doped Graphene (NPC)

Typically, 0.25 g glucose as the carbon source and 5 g dicyandiamide as the nitrogen source were dissolved in 200 mL deionized water to form a uniform solution. The solution was heated and stirred at 80°C, and the water gradually evaporated to form a powder precursor. The precursor powder was ground and mixed with an amount of triphenylphosphine and then placed into a quartz crucible. The next step is to put the crucible into the tubular furnace, anneal the quartz tube to 900°C with a rate of 3°C/min, and keep for 2 h in Ar atmosphere. Finally, N,P-doped graphene was obtained by washing and ultrasonication of the sintered black powder.

#### 4.1.3. Ru@N-Doped Graphene (Ru-NC)

Typically, 0.25 g glucose as the carbon source and 5 g dicyandiamide as the nitrogen source were dissolved in 200 mL deionized water, and then, an amount of RuCl_3_ solution was added dropwise into the mixed solution. The solution was heated and stirred at 80°C, and the water gradually evaporated to form a powder precursor. The precursor was put in a quartz crucible; then, the crucible was put in a tube furnace, annealed to 900°C at a rate of 3°C/min, and maintained in an Ar atmosphere for 2 hours. Finally, Ru@N-doped graphene was obtained by washing and ultrasonication of the sintered black powder.

#### 4.1.4. Ru@N,P-Doped Graphene (Ru-NPC)

Typically, 0.25 g glucose as the carbon source and 5 g dicyandiamide as the nitrogen source were dissolved in 200 mL deionized water, and then, an amount of RuCl_3_ solution was added dropwise into the mixed solution. The solution was heated and stirred at 80°C, and the water gradually evaporated to form a powder precursor. The precursor powder was ground and mixed with an amount of triphenylphosphine and then placed into a quartz crucible. The next step is to put the crucible into the tubular furnace, anneal the quartz tube to 900°C with a rate of 3°C/min, and keep for 2 h in the Ar atmosphere. Finally, Ru@N,P-doped graphene was obtained by washing and ultrasonication of the sintered black powder.

### 4.2. Material Characterization

Powder X-ray diffraction (XRD) studies of samples were carried out on a Philips X'Pert Pro Super X-ray diffractometer with Cu-K*α* radiation (*λ* = 1.54178 Å). The morphological studies of the samples were characterized by using JEOL JEM 2100F transmission electron microscopy (TEM) equipped with an energy-dispersive X-ray spectrometer (EDS, Oxford Instruments) for elemental mapping. Aberration-corrected high-angle annular dark-field scanning transmission electron microscopy (HAADF-STEM) measurements used a 200 kV JEM-ARM200F equipped with double spherical aberration correctors. X-ray photoelectron spectroscopy (XPS) measurements were undertaken on Thermo Fisher ESCALAB 250Xi using Al K*α* (*hν* = 1486.6 eV) as the excitation source. The binding energies of XPS spectral range were calibrated for specimen charging effects using the C 1s level at the energy of 284.6 eV as a reference. All the XPS spectra were fitted and analyzed using XPSPEAK software with a Gaussian-Lorentzian function and a nonlinear Shirley background. The N_2_ adsorption-desorption experiments were carried out using Micromeritics ASAP 2020 instrument. The Ru K-edge XAFS measurements were made at the beamline 1W1B in the Beijing Synchrotron Radiation Facility (BSRF). The energy was calibrated using a Ru metal foil for the Ru K-edge. The monochromator was detuned to reject higher harmonics. The acquired EXAFS data were processed and analyzed according to the standard procedures by using the WinXAS 3.1 program. Theoretical amplitudes and phase-shift functions were further calculated with the FEFF8.2 [39, 40]. X-ray absorption near-edge structure (XANES) calculations were carried out using the FEFF8.2 code. For the exchange correlation part of the potential, the Hedin-Lundqvist (H-L) model was employed.

### 4.3. Electrochemical Measurements

All of the electrochemical measurements were performed on the CHI760E electrochemical workstation in three-electrode systems. Typically, 5 mg of sample was first ultrasonically dispersed in 1 mL distilled water and isopropanol (volume ratio = 3 : 1) mixed solution containing 50 *μ*L of Nafion. Then, the mixed ink (5 *μ*L) was attached onto a glassy carbon rotating disk electrode with 5 mm diameter (RDE, 19.625 mm^2^) as a working electrode, a graphite rod, and Ag/AgCl (in 3 M KCl solution) were used as counter and reference electrodes, and 0.5 M H_2_SO_4_, 1 M PBS, and 1 M KOH solution were used as the electrolyte. All potentials were referenced to the reversible hydrogen electrode (RHE), and the conversion to the RHE used the Nernst equation, *E*RHE = *E*_Ag/AgCl_ + 0.059pH + *E*°_Ag/AgCl_, where *E*_Ag/AgCl_ and *E*°_Ag/AgCl_ represent the potential against the Ag/AgCl reference electrode and the standard electrode potential of Ag/AgCl at 25°C (0.197 V). Linear sweep voltammetry (LSV) was carried out with a scan rate 5 mV s^−1^ and rotating speed of 1600 r.p.m. from -0.8 to 0 V. Electrochemical impedance spectroscopy (EIS) was taken in the same configuration at open circuit voltages from 100 M to 0.1 Hz with an AC amplitude of 5 mV. The stability and durability testing were conducted by the chronopotentiometry at the current density of 10 mA cm^−2^. The Tafel plots were transformed from LSV according to the Tafel equation *η* = *b* log (*j*) + *a*, where *j* represents the current density and *b* is the Tafel slope. Cyclic voltammetry was conducted between 0.41 V and 0.51 V from 20 to 120 mV s^−1^ in acid solution, between 0.41 V and 0.51 V from 10 to 100 mV s^−1^ in neutral solution, and between 0.325 V and 0.525 V from 10 to 100 mV s^−1^ in alkaline solution, to investigate the effective surface area of the catalyst.

### 4.4. Theoretical Calculation

The structural optimization including the lattice parameters and the atomic positions is performed using VASP. The energy cutoff is 450 eV, and the Perdew-Burke-Ernzerhof (PBE) functional within the generalized gradient approximation (GGA) is adopted [41]. The van der Waals (vdW) interactions via the DFT-D2 method is used in order to properly describe the adsorption behaviors of H on the P, N, and Ru-doped graphene [42]. The free energies of the intermediates were obtained by Δ*G*(H∗) = Δ *E*(H∗) + ΔZPE‐*T*Δ*S*, where Δ*E*(H∗), *Δ*ZPE, and Δ*S* are the adsorption energy, zero point energy change, and entropy change of adsorption H, respectively. Δ*E*(H∗) was calculated by Δ*E*(H∗) = *E*(H∗+Graphene) − *E*(Graphene) − 1/2*E*(H_2_). The ZPE of H on Ru-NPC (Ru) and Ru-NPC (P) are 0.203 eV and 0.257 eV. The ZPE of H_2_ is 0.279 eV. For the vibrational entropy of two H adsorbed on the monolayer, Δ*S* = *S*(H_2_∗) − *S*(H_2_) ≈ −*S*(H_2_). For H_2_ at 300 K and 1 atm, TS(H_2_) = 0.41 eV, we have *T*Δ*S* ≈ −0.41 eV [43]. So, we have Δ*G*(H∗/P@Ru‐NPC) = Δ*E*(H∗/P@Ru‐NPC) + 0.203 − 0.279/2 − (−0.41)/2 = Δ*E*(H∗/P@Ru‐NPC) + 0.269 eV and Δ*G*(H∗/Ru@Ru‐NPC) = Δ*E*(H∗/Ru@Ru‐NPC) + 0.257 − 0.279/2 − (−0.41)/2 = Δ*E*(H∗/Ru@Ru‐NPC) + 0.323 eV.

## Supplementary Materials

Supplementary Materials Material characterization, electrochemical measurements, and theoretical calculation. Figure S1: Gibbs free energy of Ru-NC (a) and Ru-NPC (b) at P2 sites. Figure S2: the calculated electronic band structure (left) and density of states (right) of P graphene and Ru-NC. Figure S3: (a–c) the SEM images of NC at different scales and (d, e) the EDS mapping proved that atoms of C and N are uniformly distributed among the NC sample. Figure S4: (a–d) the SEM images of NPC at different scales and (e, f) the EDS mapping proved that atoms of C, N, and P are uniformly distributed among the NPC sample. Figure S5: (a–d) the SEM images of Ru-NC at different scales and (e–g) the EDS mapping proved that atoms of C, N, and Ru are uniformly distributed among the Ru-NC sample. Figure S6: (a–c) the SEM images of Ru-NPC at different scales and (d–g) the EDS mapping proved that atoms of C, N, P, and Ru are uniformly distributed among the Ru-NPC sample. Figure S7: N_2_ adsorption and desorption isotherms of NC, NPC, Ru-NC, and Ru-NPC. Figure S8: HADDF-STEM images of Ru-NPC at a different scale. Figure S9: (a, b) the TEM and HRTEM images of Ru-NC show a typical two-dimensional graphene structure with no impurity particles on the surface. (c) The local high resolution STEM image shows that Ru-NC displays a random dispersion of Ru single atoms on the nanosheets. (g) The EDS mapping proved that atoms of C, N, and Ru are uniformly distributed among the Ru-NC sample. Figure S10: (a) XPS spectra of C 1s binding energy. (b) XPS spectra of N 1s binding energy with data fitting. Figure S11: (a) XPS spectra of C 1s binding energy. (b) XPS spectra of N 1s binding energy. (c) XPS spectra of P 2p binding energy with data fitting. Figure S12: (a) XPS spectra of C 1s binding energy. (b) XPS spectra of N 1s binding energy. (c) XPS spectra of Ru 3p binding energy with data fitting. Figure S13: (a) XPS spectra of C 1s binding energy. (b) XPS spectra of N 1s binding energy. (c) XPS spectra of P 2p binding energy. (c) XPS spectra of Ru 3p binding energy with data fitting. Figure S14: XPS spectra of Ru 3p for Ru-NC and Ru-NPC. (b) The XANES spectra of Ru-NC, Ru-NPC, RuO_2_, and Ru foil. Figure S15: the fitting curve of Ru K-edge in Ru-NC and Ru-NPC. Figure S16: HADDF-STEM images of Ru-NPC after CVs for 3000 cycles in acidic solution. Figure S17: (a–d) Cyclic voltammograms of NC, NPC, Ru-NC, and Ru-NPC catalysts at different scan rates in acidic solution. (e) Double-layer capacitance measurements for determining electrochemically active surface areas of NC, NPC, Ru-NC, and Ru-NPC catalysts. Figure S18: (a–d) cyclic voltammograms of NC, NPC, Ru-NC, and Ru-NPC catalysts at different scan rates in alkaline solution. (e) Double-layer capacitance measurements for determining electrochemically active surface areas of NC, NPC, Ru-NC, and Ru-NPC catalysts. Figure S19: (a–d) cyclic voltammograms of NC, NPC, Ru-NC, and Ru-NPC catalysts at different scan rates in neutral solution. (e) Double-layer capacitance measurements for determining electrochemically active surface areas of NC, NPC, Ru-NC, and Ru-NPC catalysts. Figure S20: comparative electrocatalytic hydrogen evolution of catalysts. (a) HER polarization curves of different samples and commercial 20% Pt/C catalyst in 1 M KOH solution. (b) Corresponding Tafel plots of different samples and commercial 20% Pt/C catalyst in 1 M KOH solution. (c) HER polarization curves of Ru-NPC before and after 3000 CV tests (inset: time dependence of cathodic current density curve (*v* − *t* curve) of Ru-NPC) in 1 M KOH solution. Figure S21: the in situ synchrotron radiation infrared spectra of Ru-NC and Ru-NPC before HER. Figure S22: the stability curve of the *I*‐*t* test at -0.1 V (vs. RHE) of the Ru-NPC sample in situ synchrotron radiation infrared spectroscopy test. Figure S23: the in situ synchrotron radiation infrared spectra of the Ru-NC catalyst at different test times. Table S1: local structural parameters for the Ru atom in Ru-NC, Ru-NPC, and Ru foil fitted from EXAFS data. Table S2: comparison of electrocatalytic HER activity about single-atom catalysts.
