# Supplementary material for: A Unique Ru-N4-P Coordinated Structure Synergistically Waking Up the Nonmetal P Active Site for Hydrogen Production
Source: Research (Wash D C). 2020 Aug 28;2020:5860712. doi: 10.34133/2020/5860712 (PMC7521024; doi:10.34133/2020/5860712)
Supplement: Supplementary Materials — Material characterization, electrochemical measurements, and theoretical calculation. Figure S1: Gibbs free energy of Ru-NC (a) and Ru-NPC (b) at P2 sites. Figure S2: the calculated electronic band structure (left) and density of states (right) of P graphene and Ru-NC. Figure S3: (a–c) the SEM images of NC at different scales and (d, e) the EDS mapping proved that atoms of C and N are uniformly distributed among the NC sample. Figure S4: (a–d) the SEM images of NPC at different scales and (e, f) the EDS mapping proved that atoms of C, N, and P are uniformly distributed among the NPC sample. Figure S5: (a–d) the SEM images of Ru-NC at different scales and (e–g) the EDS mapping proved that atoms of C, N, and Ru are uniformly distributed among the Ru-NC sample. Figure S6: (a–c) the SEM images of Ru-NPC at different scales and (d–g) the EDS mapping proved that atoms of C, N, P, and Ru are uniformly distributed among the Ru-NPC sample. Figure S7: N2 adsorption and desorption isotherms of NC, NPC, Ru-NC, and Ru-NPC. Figure S8: HADDF-STEM images of Ru-NPC at a different scale. Figure S9: (a, b) the TEM and HRTEM images of Ru-NC show a typical two-dimensional graphene structure with no impurity particles on the surface. (c) The local high resolution STEM image shows that Ru-NC displays a random dispersion of Ru single atoms on the nanosheets. (g) The EDS mapping proved that atoms of C, N, and Ru are uniformly distributed among the Ru-NC sample. Figure S10: (a) XPS spectra of C 1s binding energy. (b) XPS spectra of N 1s binding energy with data fitting. Figure S11: (a) XPS spectra of C 1s binding energy. (b) XPS spectra of N 1s binding energy. (c) XPS spectra of P 2p binding energy with data fitting. Figure S12: (a) XPS spectra of C 1s binding energy. (b) XPS spectra of N 1s binding energy. (c) XPS spectra of Ru 3p binding energy with data fitting. Figure S13: (a) XPS spectra of C 1s binding energy. (b) XPS spectra of N 1s binding energy. (c) XPS spectra of P 2p binding ener [file 5860712.f1.zip › SI.docx]

**A Unique Ru-N_4_-P Coordinated Structure Synergistically Waking up the Non-metal P Active Site for Hydrogen Production**

Chuanqiang Wu^1,4†^, Shiqing Ding^1†^, Daobin Liu^1†^, Dongdong Li^2†^, Shuangming Chen^1*^, Huijuan Wang^3^, Zeming Qi^1^, Binghui Ge^4^, Li Song^1*^

^1^ National Synchrotron Radiation Laboratory, CAS Center for Excellence in Nanoscience, University of Science and Technology of China, Hefei, Anhui 230029, P. R. China.

^2^ Institute of Amorphous Matter Science, School of Materials Science and Engineering, Hefei University of Technology, Hefei, Anhui 230009, P. R. China.

^3^ Experimental Center of Engineering and Material Science, University of Science and Technology of China, Hefei, 230026, P. R. China.

^4^ Institutes of Physical Science and Information Technology, Anhui University, Hefei 230601, P. R. China.

CORRESPONDING AUTHOR:

Shuangming Chen*: csmp@ustc.edu.cn; Li Song*: song2012@ustc.edu.cn

**KEYWORDS:** Nonmetallic active site, Ru-N_4_-P structure, Monoatomic HER catalyst, XAFS spectrum, In situ infrared spectrum

**
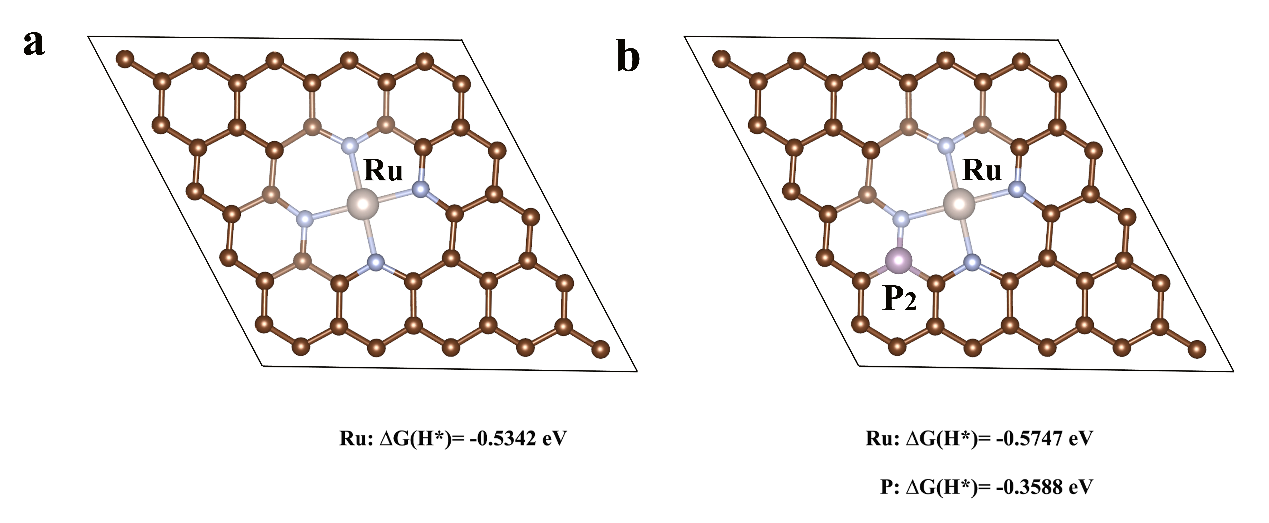
Figure S1.** Gibbs free energy of Ru-NC (a) and Ru-NPC (b) at P_2_ sites.


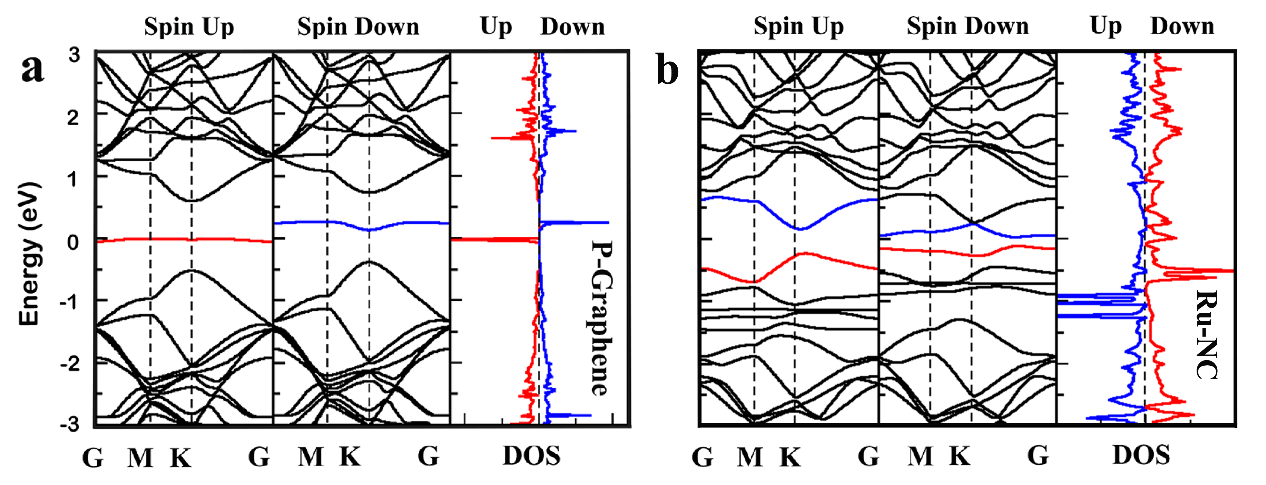


**Figure S2.** The calculated electronic band structure (left) and density of states (right) of P-graphene and Ru-NC.


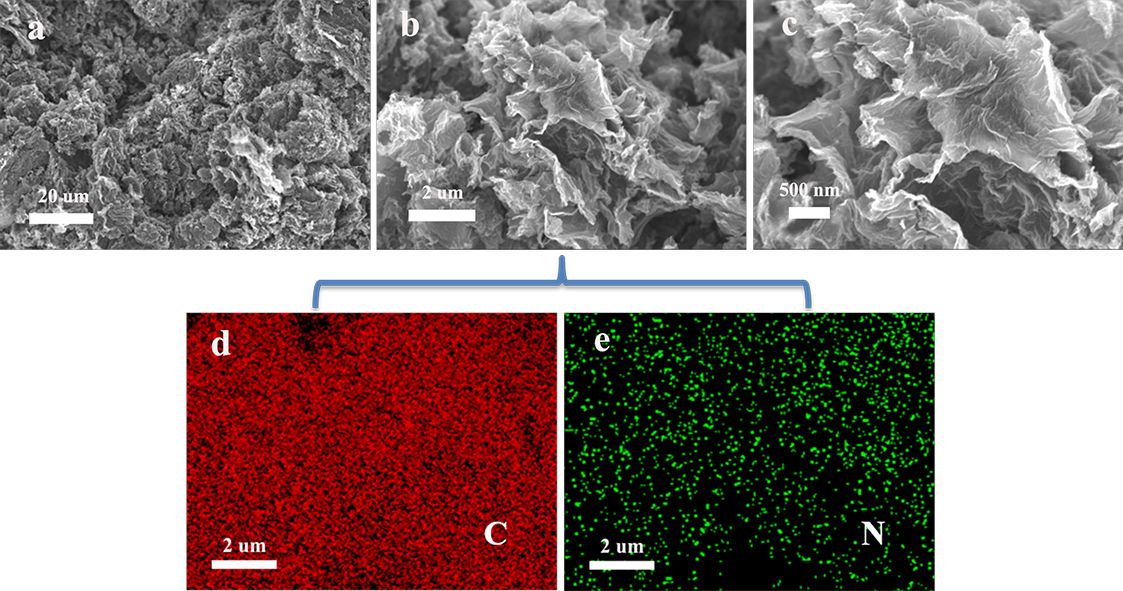


**Figure S3.** (a, b, c) The SEM images of NC at different scales and (d, e) The EDS mapping proved that atoms of C and N uniformly distributed among the NC sample.


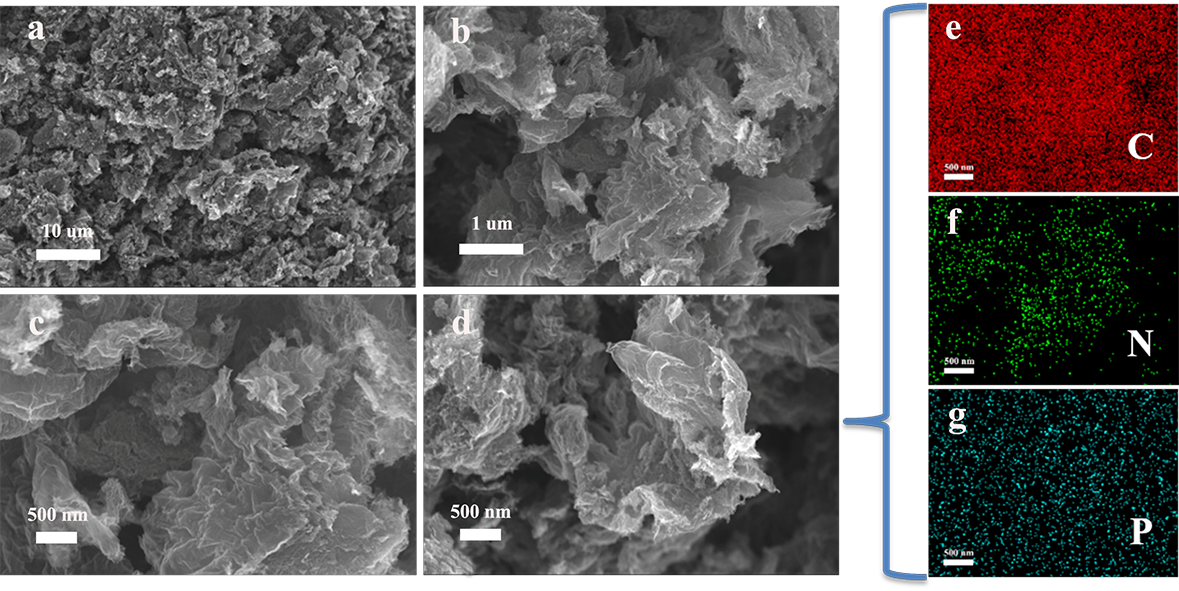


**Figure S4.** (a, b, c, d) The SEM images of NPC at different scales and (e, f) The EDS mapping proved that atoms of C, N and P are uniformly distributed among the NPC sample.


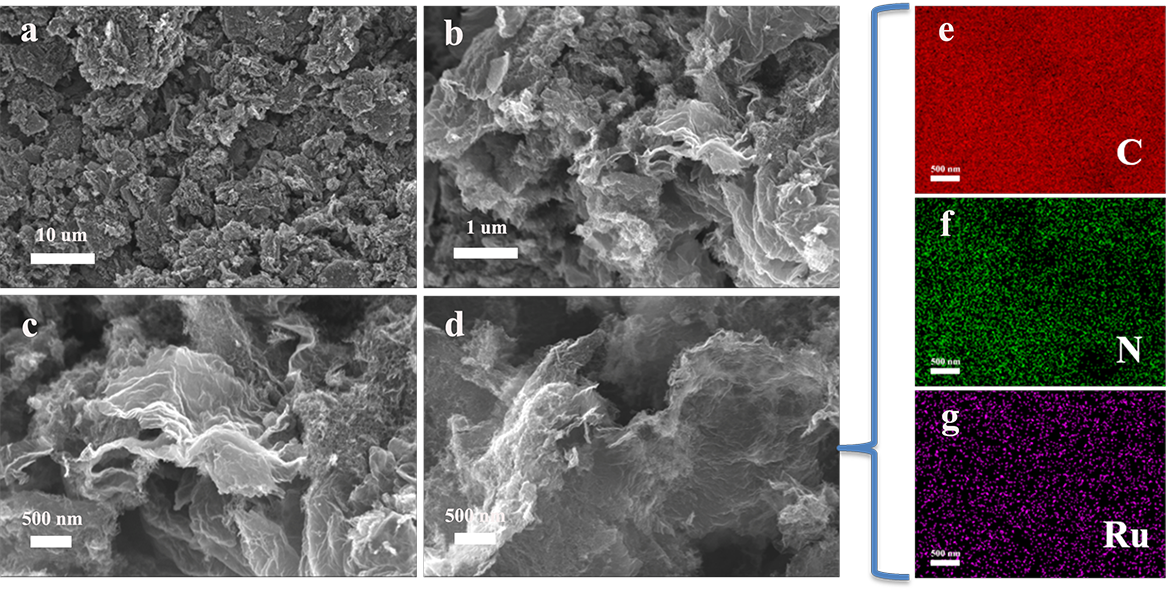


**Figure S5.** (a, b, c, d) The SEM images of Ru-NC at different scales and (e, f, g) The EDS mapping proved that atoms of C, N and Ru are uniformly distributed among the Ru-NC sample.


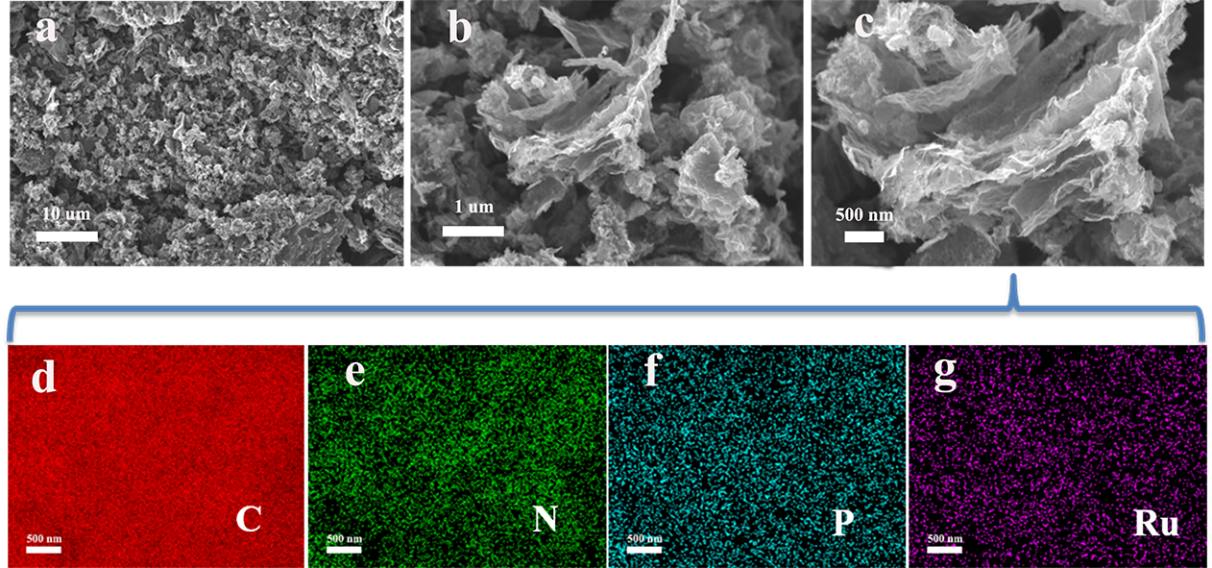


**Figure S6.** (a, b, c) The SEM images of Ru-NPC at different scales and (d, e, f, g) The EDS mapping proved that atoms of C, N, P and Ru are uniformly distributed among the Ru-NPC sample.


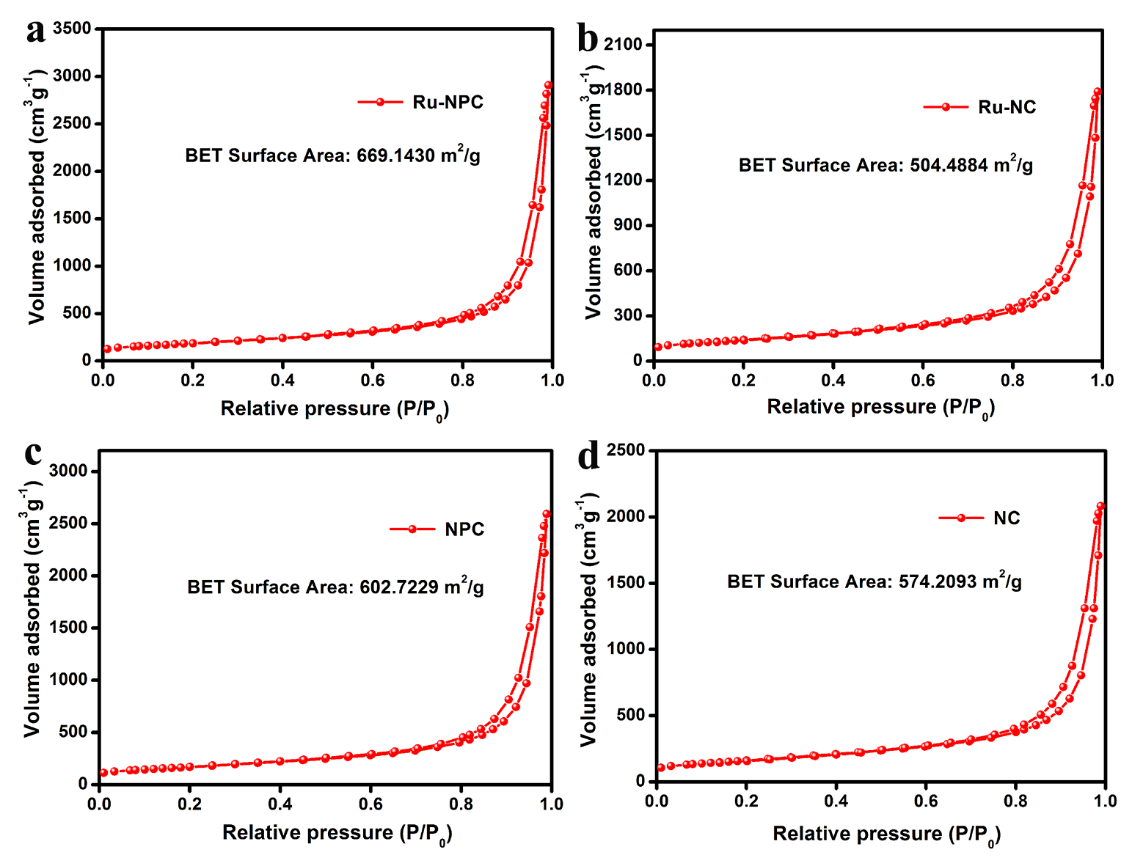


**Figure S7.** N_2_ adsorption and desorption isotherms of NC, NPC, Ru-NC and Ru-NPC.


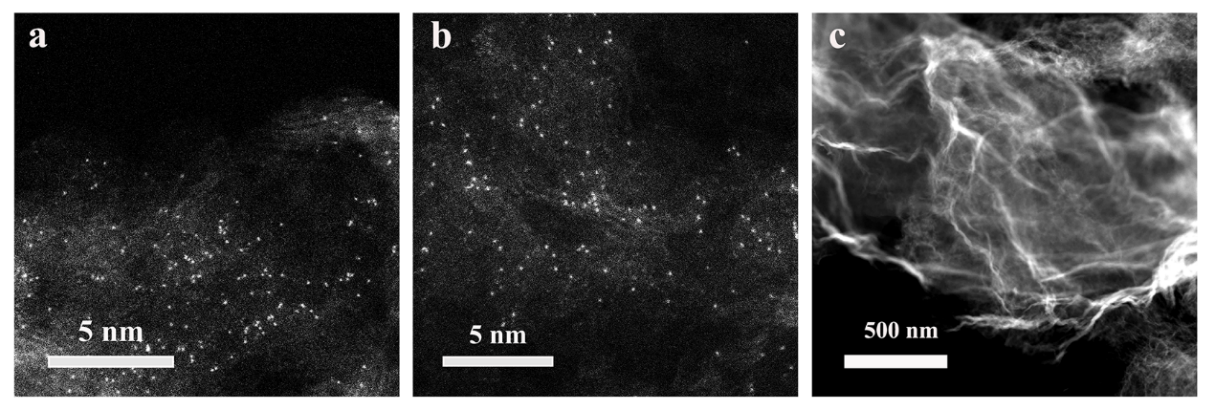


**Figure S8.** HADDF-STEM images of Ru-NPC at a different scale.


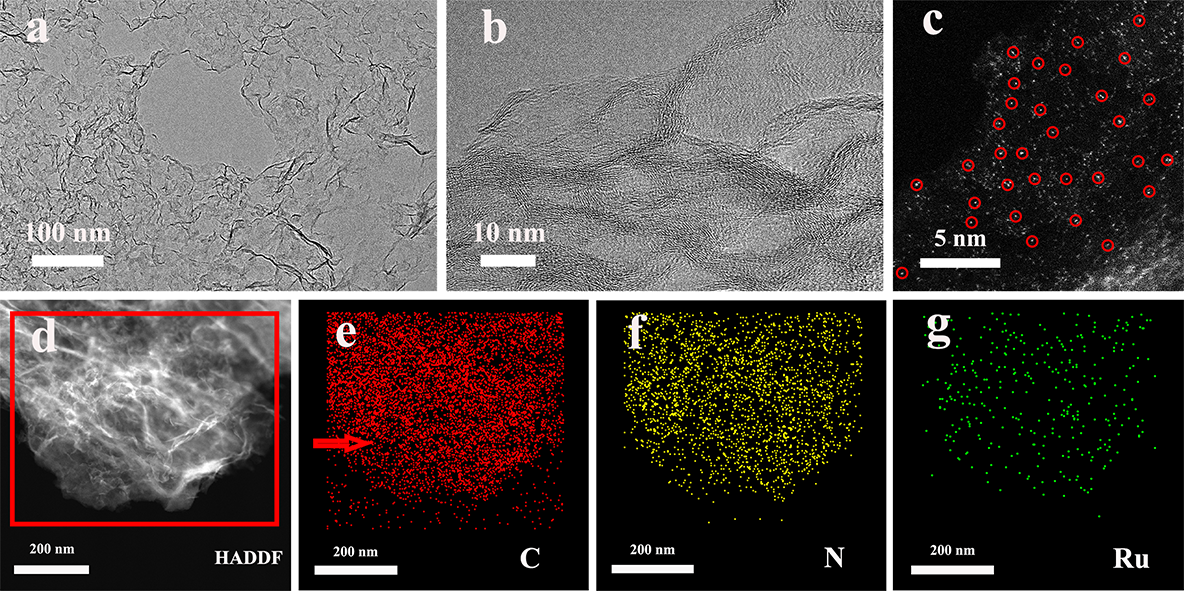


**Figure S9.** (a, b) The TEM and HRTEM images of Ru-NC show a typical two-dimensional graphene structure with no impurity particles on the surface. (c) The local high resolution STEM image shows that Ru-NC displays a random dispersion of Ru single atoms on the nanosheets. (g) The EDS mapping proved that atoms of C, N and Ru are uniformly distributed among the Ru-NC sample.


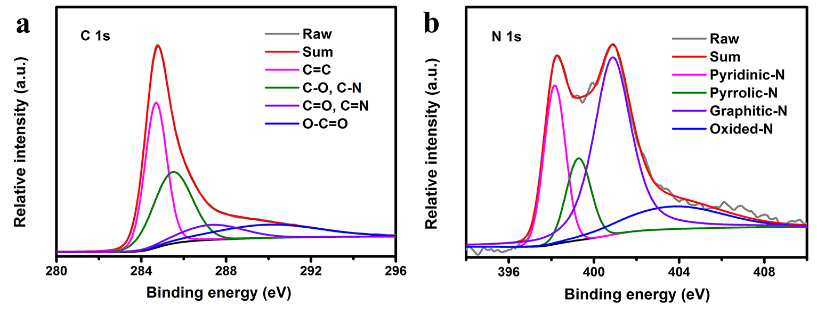


**Figure S10.** (a) XPS spectra of C 1s binding energy. (b) XPS spectra of N 1s binding energy with data fitting.


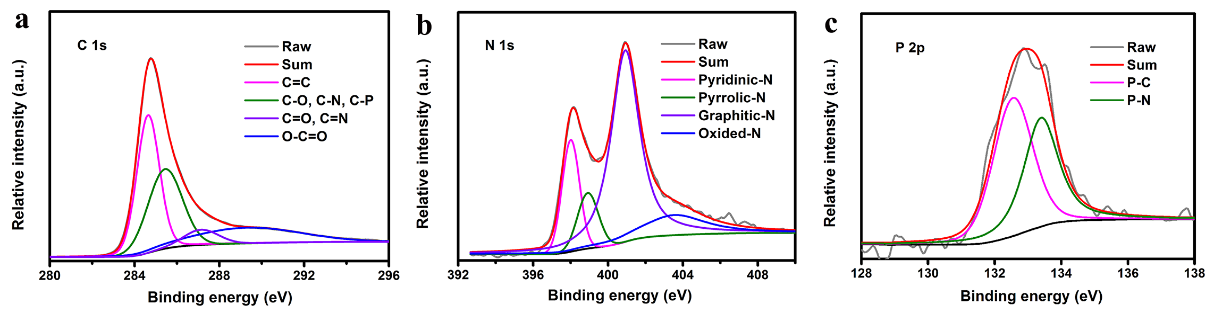


**Figure S11.** (a) XPS spectra of C 1s binding energy. (b) XPS spectra of N 1s binding energy. (c) XPS spectra of P 2p binding energy with data fitting.


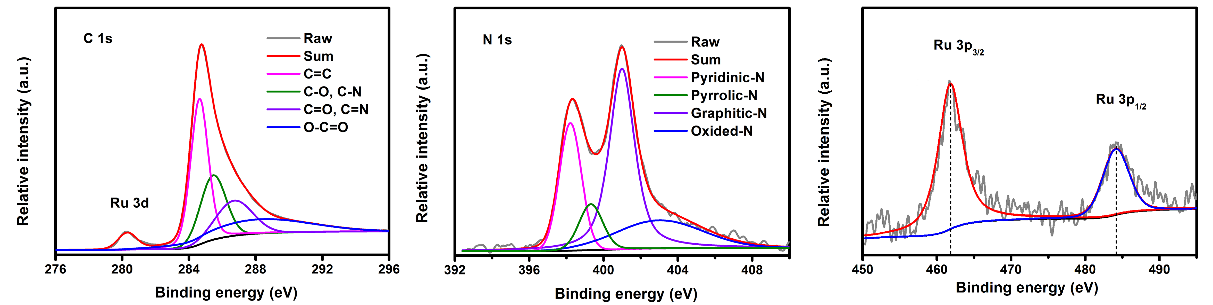


**Figure S12.** (a) XPS spectra of C 1s binding energy. (b) XPS spectra of N 1s binding energy. (c) XPS spectra of Ru 3p binding energy with data fitting.


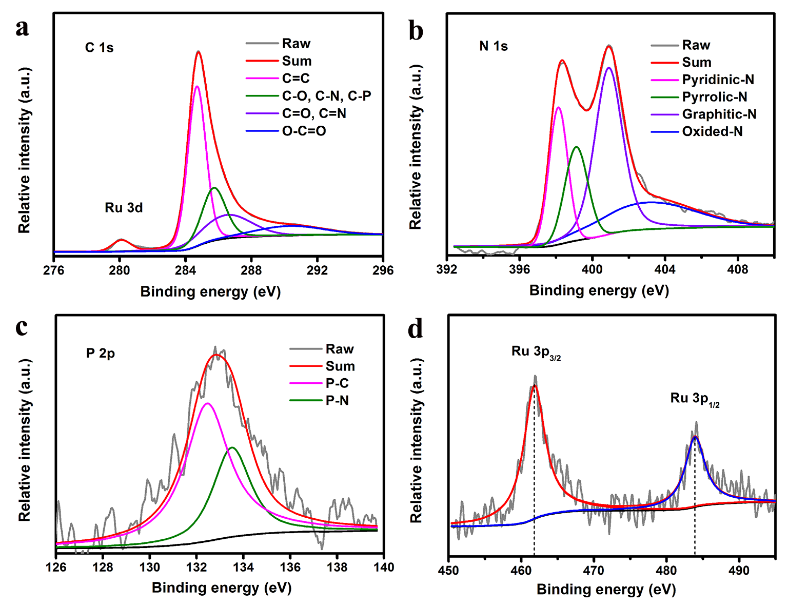


**Figure S13.** (a) XPS spectra of C 1s binding energy. (b) XPS spectra of N 1s binding energy. (c) XPS spectra of P 2p binding energy. (c) XPS spectra of Ru 3p binding energy with data fitting


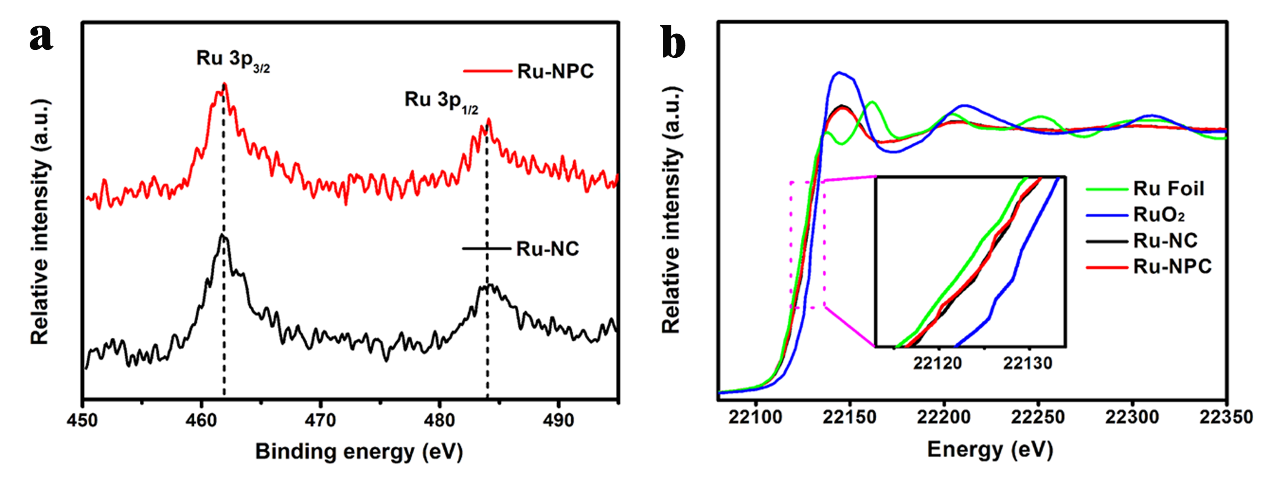


**Figure S14**. XPS spectra of Ru 3p for Ru-NC and Ru-NPC. (b) The XANES spectra of Ru-NC, Ru-NPC, RuO_2_ and Ru foil.


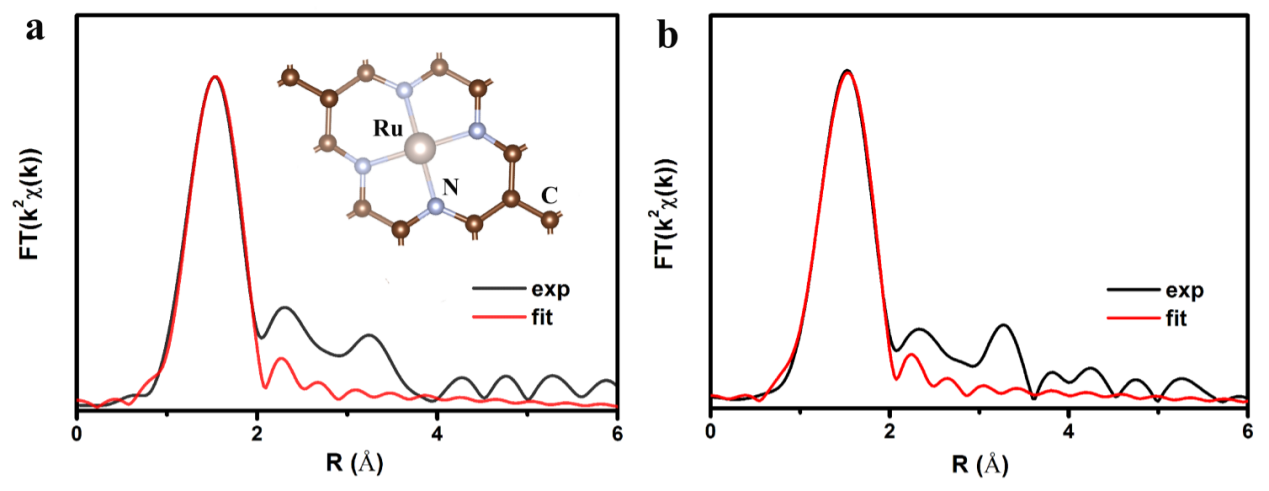


**Figure S15**. The fitting curve of Ru K-edge in Ru-NC and Ru-NPC.


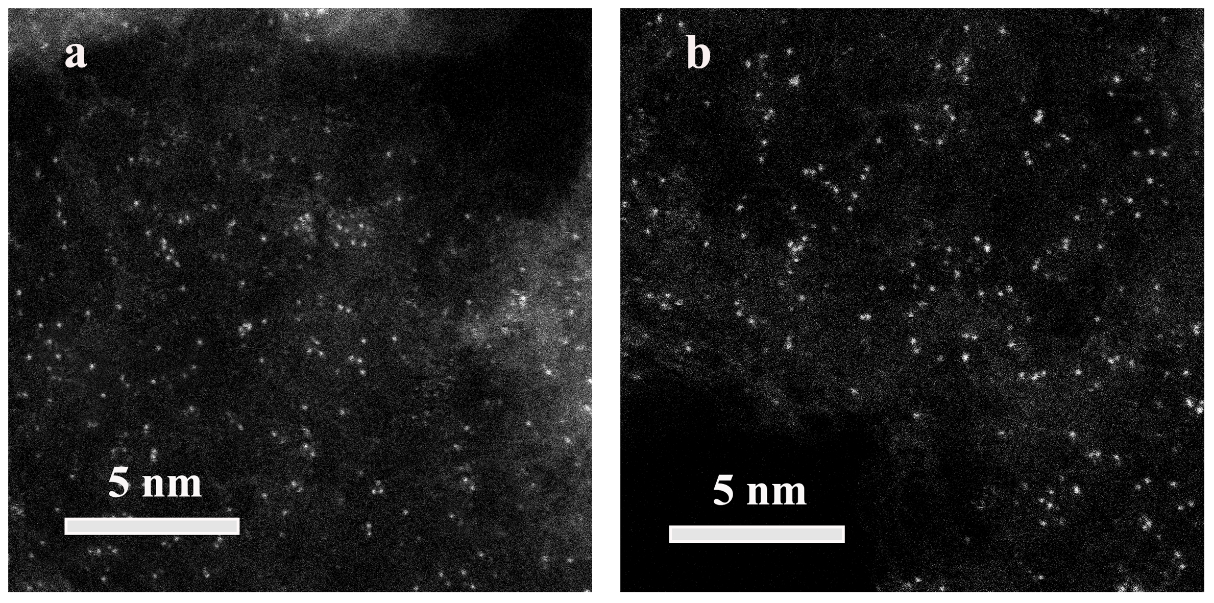


**Figure S16.** HADDF-STEM images of Ru-NPC after CVs for 3000 cycles in acidic solution.


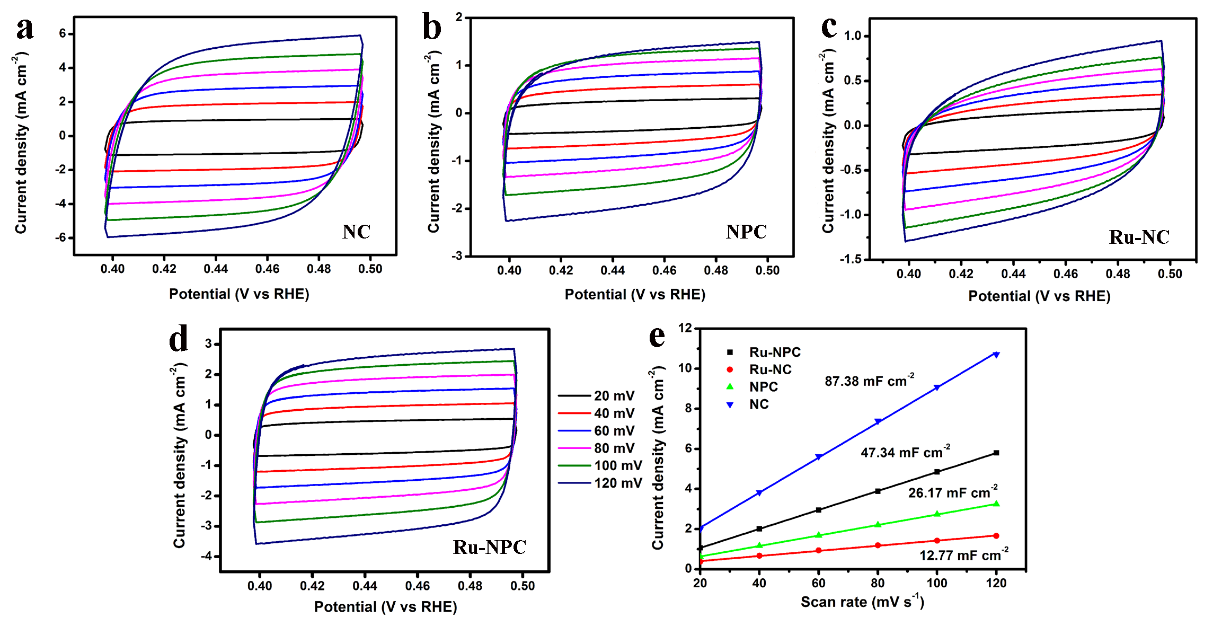


**Figure S17.** (a-d) Cyclic voltammograms of NC, NPC, Ru-NC and Ru-NPC catalysts at different scan rate in acidic solution. (e) Double-layer capacitance measurements for determining electrochemically active surface areas of NC, NPC, Ru-NC and Ru-NPC catalysts.


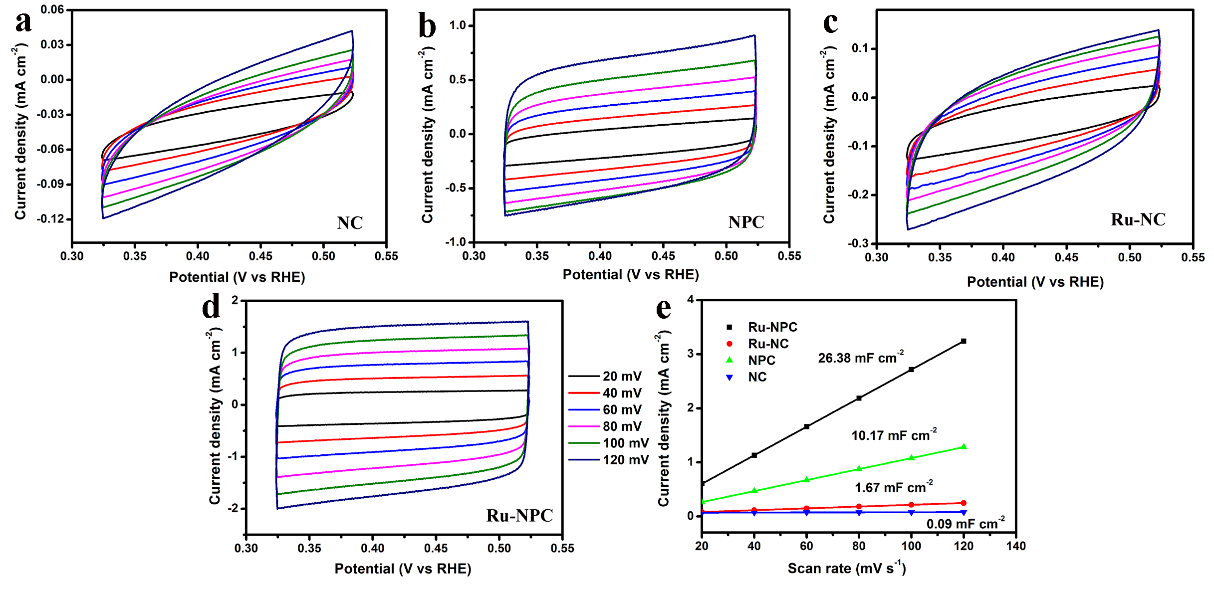


**Figure S18.** (a-d) Cyclic voltammograms of NC, NPC, Ru-NC and Ru-NPC catalysts at different scan rate in alkaline solution. (e) Double-layer capacitance measurements for determining electrochemically active surface areas of NC, NPC, Ru-NC and Ru-NPC catalysts.


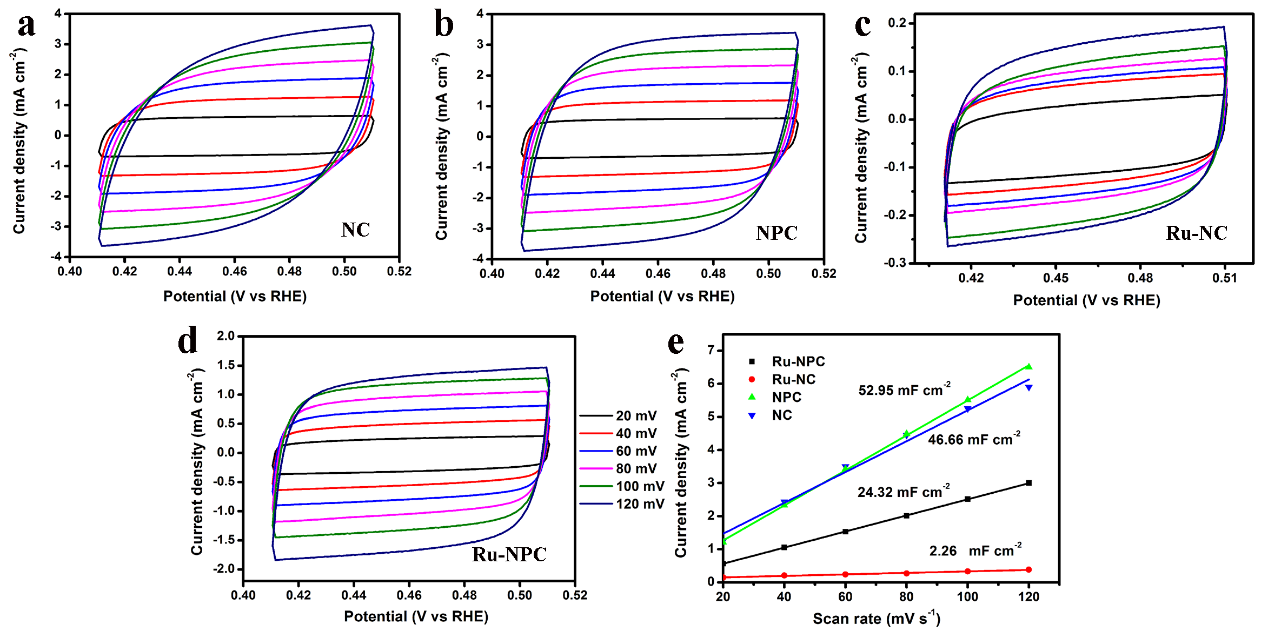


**Figure S19.** (a-d) Cyclic voltammograms of NC, NPC, Ru-NC and Ru-NPC catalysts at different scan rate in neutral solution. (e) Double-layer capacitance measurements for determining electrochemically active surface areas of NC, NPC, Ru-NC and Ru-NPC catalysts.


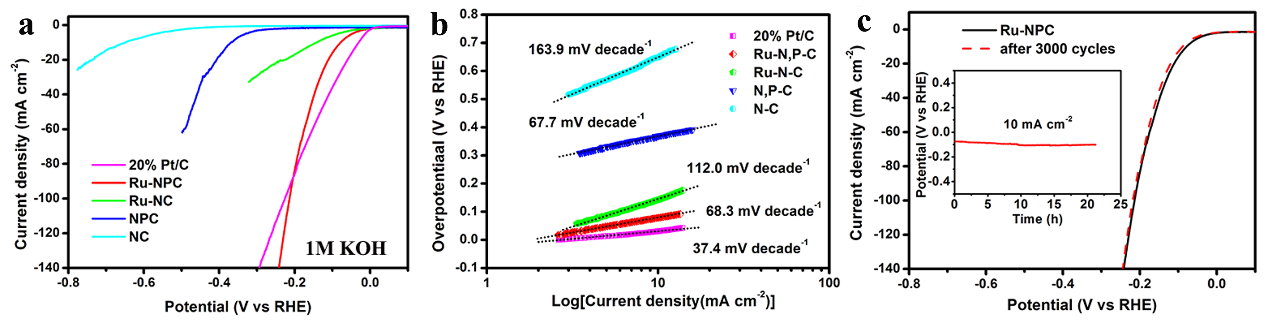


**Figure S20.** Comparative electrocatalytic hydrogen evolution of catalysts. (a) HER polarization curves of different samples and commercial 20% Pt/C catalyst in 1 M KOH solution. (b) Corresponding Tafel plots of different samples and commercial 20% Pt/C catalyst in 1 M KOH solution. (c) HER polarization curves of Ru-NPC before and after 3000 CV tests (inset: time-dependence of cathodic current density curve (v−t curve) of Ru-NPC) in 1 M KOH solution.


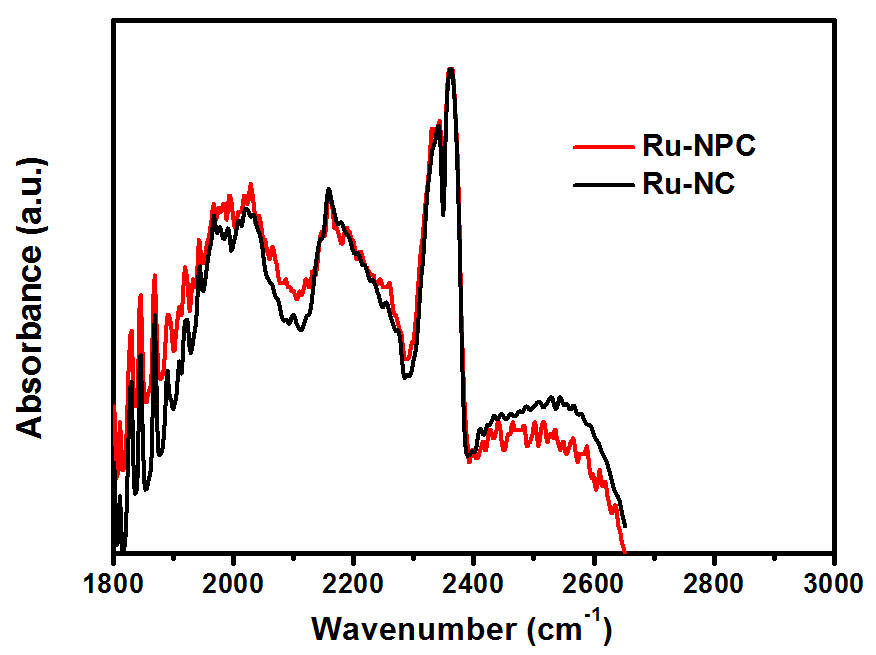


**Figure S21.** The in situ synchrotron radiation infrared spectra of Ru-NC and Ru-NPC before HER.


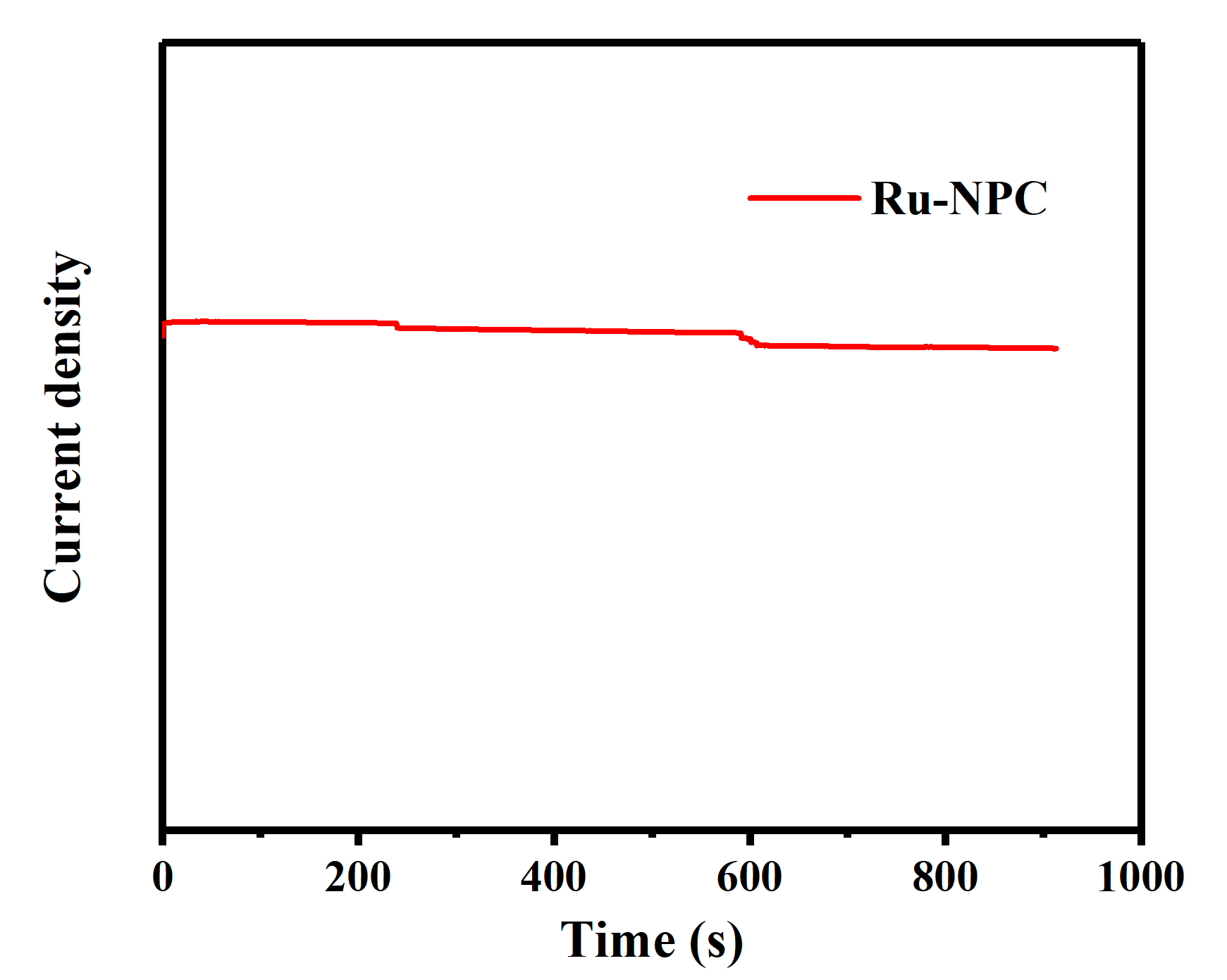


**Figure S22.** The stability curve of the I-t test at -0.1 V (vs RHE) of the Ru-NPC sample in situ synchrotron radiation infrared spectroscopy test.


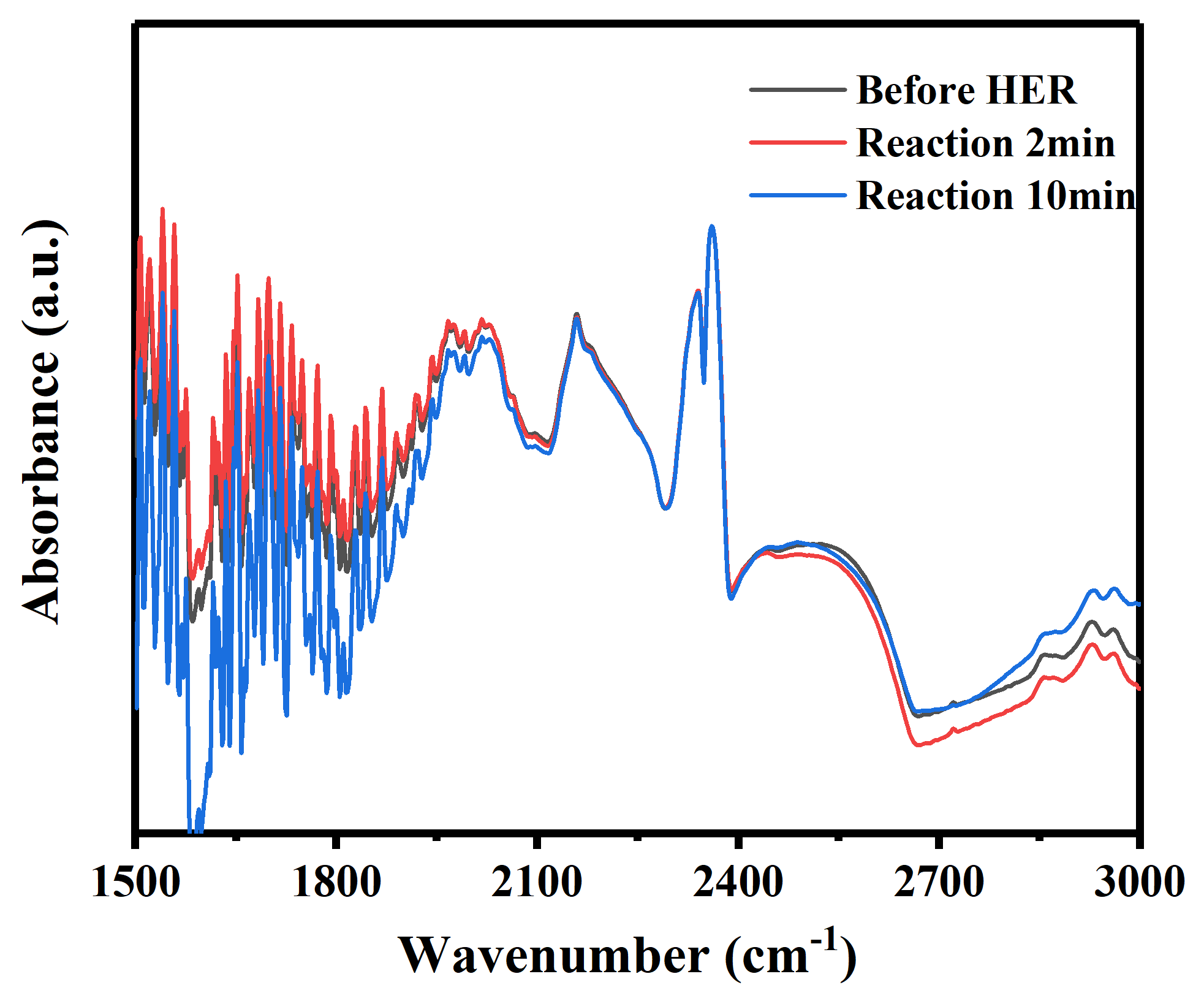


**Figure S23.** The in situ synchrotron radiation infrared spectra of the Ru-NC catalyst at different test times.

**Table S1.** Local structural parameters for the Ru atom in Ru-NC, Ru-NPC and Ru foil fitted from EXAFS data.

| **Sample** | **Bond** | **N** | **Bond length** | **σ^2^(10^-3^ Å^2^)** |
| --- | --- | --- | --- | --- |
| **Ru-NC** | Ru-N | 3.7 | 2.06 | 4.1 |
| **Ru-NPC** | Ru-N | 3.8 | 2.05 | 4.4 |
| **Ru foil** | Ru-Ru | 12* | 2.68 | 2.7 |

N, coordination number; σ^2^, Debye-Waller factor; Error bounds (accuracies) were estimated as N, ±10%; Bond length, ±1%; σ^2^, ±10%. * is fixed coordination number according to the standard crystal structure.

**Table S2.** Comparison of electrocatalytic HER activity about single atoms catalysts.

| **Catalys** | **Tafel slope (mV/des)a** | **Overpential at 10 mA/cm^2^** | **Condition** | **Reference** |
| --- | --- | --- | --- | --- |
| W-SAC-MOF | 53.0 | -85 mV | 0.1 M KOH | Adv. Mater. 2018, 30, 1800396. |
| Co-N-P-CNFs | 56.0 | -248 mV | 0.5 M H_2_SO_4_ | J Power Sources. 2016, 311, 68-80. |
| Ru-SAs-HPN matrix | 38.0 | -24 mV | 0.5 M H_2_SO_4_ | Angew. Chem. Int. Ed. 2018, 57, 9495-9500. |
| Pt-Mo_2_TiC_2_T_x_ | 30.0 | -30 mV | 0.5 M H_2_SO_4_ | Nat. Catal. 2018, 1, 985-992. |
| Pt-OLC catalyst | 36.0 | -38 mV | 0.5 M H_2_SO_4_ | Nat. Energ. 2019. 4. 512-518. |
| Pt SASs/AG | 30.0 | -12 mV | 0.5 M H_2_SO_4_ | Energy Environ. Sci., 2019, 12, 1000-1007. |
| Pt-CoP-Ni foam | 30.0 | -24 mV | 1 M PBS | Angew. Chem. Int. Ed. 2017, 56, 13694-13698. |
| Graphene-analogous | 44.6 | -57 mV | 0.5 M H_2_SO_4_ | Angew. Chem. Int. Ed. 2019, 58, 2-10. |
| SANi-I | 34.6 | -60 mV@100 mA  cm^-2^ | 0.1 M KOH | Angew. Chem. Int. Ed. DOI: 10.1002/anie.201908210 |
| Pt single atoms @ MoS_2_ | 96.0 | -130 mV | 0.1 M H_2_SO_4_ | Energy Environ. Sci., 2015, 8, 1594-1601. |
| ALD50Pt/NGNs | 29.0 | -50 mV@16 mA  cm^-2^ | 0.5 M H_2_SO_4_ | Nat. Commun. 2016. 7. 13638. |
| Ru-NPC | 59.4 | 93 | 0.5 M H_2_SO_4_ | This Work |
|  | 68.3 | 78 | 1 M KOH |  |
